# Comparative efficacy and safety of biologic therapies in pediatric asthma: a comprehensive systematic review

**DOI:** 10.3389/fmed.2026.1722577

**Published:** 2026-03-18

**Authors:** Abdullah Alzayed

**Affiliations:** Department of Pediatrics, College of Medicine, Imam Mohammad Ibn Saud Islamic University (IMSIU), Riyadh, Saudi Arabia

**Keywords:** asthma, asthma exacerbation safety, biologic therapy, pediatrics, quality of life

## Abstract

**Background:**

Asthma remains one of the most prevalent chronic diseases among children, with severe cases posing significant challenges to symptom control, and quality of life (QoL). Biologic therapies have emerged as effective alternatives for severe asthma unresponsive to conventional therapies. However, limited evidence exists for their comparative efficacy and safety in pediatric populations. This systematic review aims to evaluate the efficacy and safety of five major biologic agents in children and adolescents with uncontrolled asthma.

**Methods:**

A systematic literature search was conducted in PubMed, Web of Science, and Embase up to June 2025. Eligible studies included randomized and open label extensions involving pediatric asthma patients treated with at least one of the five biologic therapies including Dupilumab, Omalizumab, Mepolizumab, Lebrikizumab and Benralizumab. Outcomes assessed included exacerbation rates, lung function, asthma control, QoL, and adverse events. Risk of bias was assessed using RoB 2.0 and MINORS tools.

**Results:**

This review included Twenty-five studies. Dupilumab consistently showed the most robust outcomes, significantly reducing exacerbations (by up to 64.7%), improving ACQ-7 scores, pulmonary function, and QoL. Omalizumab also showed reduced exacerbations and improved symptom control and QoL, with debatable results in pulmonary function. Mepolizumab demonstrated moderate benefits with variable efficacy and higher SAE rates. Benralizumab and Lebrikizumab yielded modest improvements in clinical outcomes. Safety profiles were generally favorable across biologics, with mild-to-moderate adverse events.

**Conclusion:**

Among the reviewed biologics, Dupilumab showed the most consistent and sustained efficacy across clinical, and patient-reported outcomes in pediatric asthma. Omalizumab also proved effective, particularly in allergic and virus-induced exacerbations. This review underscores the importance of phenotype-directed therapy and supports dupilumab as a preferred option for long-term management of severe pediatric asthma.

## 1 Introduction

Asthma is a chronic, heterogeneous respiratory disease characterized by airway inflammation, variable airflow obstruction, and bronchial hyperresponsiveness (1). It affects an estimated 262 million people worldwide and results in nearly 455,000 deaths annually, according to the World Health Organization (2). Its incidence and prevalence have been rising globally, particularly in urbanized regions and low to middle income countries, caused by environmental exposures, genetic predisposition, allergens, air pollution, and lifestyle factors (3). Risk factors for asthma include a family history of atopy or asthma, exposure to tobacco smoke (especially during pregnancy and early childhood), respiratory infections, and occupational irritants (4). Vulnerable populations, such as children, the elderly, and those with low socioeconomic status, bear a disproportionate burden of disease due to limited access to care and environmental exposures (5).

Asthma in children is of particular concern due to its impact on growth, school attendance, quality of life, and long-term pulmonary development. Pediatric asthma is often triggered by allergens, viral infections, and environmental pollutants, and it can range from mild intermittent to severe persistent disease (6). Pediatric asthma represents a major public health burden, accounting for millions of emergency department visits and hospitalizations annually worldwide, and is one of the leading causes of school absenteeism. In the United States alone, pediatric asthma is responsible for billions of dollars annually in direct and indirect healthcare costs, largely driven by hospitalizations and exacerbation-related care. Poorly controlled asthma in children can lead to frequent exacerbations, hospitalizations, and a high burden on families and healthcare systems (6). Standard treatment approaches include inhaled corticosteroids (ICS), long-acting beta-agonists, leukotriene receptor antagonists, and oral corticosteroids in severe cases (7).

Biologic therapies represent a major advancement in the management of severe asthma by targeting specific immunological pathways involved in the disease. These therapies are administered via subcutaneous or intravenous routes and are primarily indicated for moderate-to-severe asthma that is uncontrolled despite optimized standard treatment (8).

Omalizumab was the first biologic approved for asthma and is a recombinant humanized monoclonal antibody that targets immunoglobulin E (IgE) (9). It binds to free IgE, preventing it from interacting with its high-affinity receptor (FcεRI) on mast cells and basophils, thereby reducing allergic inflammation. Omalizumab has demonstrated efficacy in reducing exacerbation rates, improving quality of life, and reducing corticosteroid use in patients with allergic asthma. However, rare complications such as anaphylaxis and injection-site reactions may occur (7, 9).

Mepolizumab, a monoclonal antibody targeting interleukin-5 (IL-5), prevents IL-5 from binding to its receptor on eosinophils, thereby reducing eosinophilic inflammation, which plays a central role in severe asthma. Clinical trials have shown that mepolizumab significantly reduces exacerbations, improves lung function (FEV1), and lowers oral corticosteroid dependence in patients with eosinophilic asthma. Adverse effects are generally mild, with headache and injection site reactions being most common (8, 10).

Benralizumab is another IL-5 pathway-targeting monoclonal antibody but acts by binding to the IL-5 receptor *α* (IL-5Rα) on eosinophils and basophils, inducing antibody-dependent cell-mediated cytotoxicity, leading to rapid and near-complete depletion of eosinophils (11). Its mechanism offers a faster and more sustained reduction of eosinophilic inflammation. Evidence from phase III trials demonstrates substantial reductions in asthma exacerbations, improvements in asthma control, and decreased reliance on systemic steroids. Common side effects include nasopharyngitis and headache, though serious adverse effects are rare (8, 11).

Dupilumab is a fully human monoclonal antibody that blocks the shared IL-4 receptor alpha (IL-4Rα) subunit, inhibiting both IL-4 and IL-13 signaling, which are key drivers of Type 2 inflammation. Type 2 asthma refers to asthma characterized by eosinophilic inflammation and/or elevated biomarkers such as blood eosinophils, fractional exhaled nitric oxide (FeNO), and IgE, driven primarily by IL-4, IL-5, and IL-13 pathways. It is approved for moderate-to-severe eosinophilic asthma and asthma with oral corticosteroid dependency (12). Dupilumab has shown efficacy in improving lung function, asthma control scores, and reducing exacerbations, with added benefits in comorbid conditions such as atopic dermatitis and chronic rhinosinusitis with nasal polyps. Its side effects include injection-site reactions, conjunctivitis, and transient eosinophilia (8, 12).

To our knowledge, this is the first comprehensive systematic review that aims to evaluate and compare the efficacy, safety, and clinical outcomes of all five major biologic agents Dupilumab, Omalizumab, Mepolizumab, lebrikizumab and Benralizumab for asthma control. While biologic therapies have been extensively studied in adult asthma, comparative evidence in pediatric populations remains limited. This systematic review specifically focuses on children and adolescents with asthma, aiming to synthesize pediatric-specific efficacy and safety data across currently available biologic therapies.

## 2 Methods

This systematic review was designed in accordance with the Preferred Reporting Items for Systematic Reviews and Meta-Analyses (PRISMA) 2020 guidelines (13).

### 2.1 Inclusion and exclusion criteria

We included studies that met the following predefined eligibility criteria. We included studies that met the following predefined eligibility criteria. Participants were primarily children and adolescents diagnosed with mild to severe asthma. Studies including mixed populations (adolescents and adults) were eligible only if pediatric data were reported separately, if adolescents constituted a predefined subgroup, or if the study represented an extension of a pediatric clinical trial. Adult-only studies were excluded from the qualitative synthesis and were not used to inform the primary conclusions. Interventions were limited to one or more of the following biologic therapies: Dupilumab, Omalizumab, Mepolizumab, lebrikizumab or Benralizumab. These were compared to either placebo or standard therapy (e.g., inhaled corticosteroids, long-acting beta-agonists). Only randomized and non-randomized controlled trials published in English and reporting on relevant efficacy and safety outcomes were included. Studies were excluded if they were reviews, editorials, commentaries, conference abstracts, animal studies, or lacked extractable outcome data.

### 2.2 Search strategy

We conducted a systematic search of various electronic databases: PubMed, Web of Science, and Embase, from inception until June 2025. The search terms included a combination of MeSH terms and free-text keywords such as “asthma,” “biologic therapy,” “Dupilumab,” “Omalizumab,” “Mepolizumab,” “lebrikizumab,” “Benralizumab” and “randomized controlled trial,”. For full search strategy see Supplementary file 1.

### 2.3 Screening

First all titles and abstracts were screened for relevance. Full texts of potentially eligible articles were retrieved and assessed against the inclusion criteria.

### 2.4 Data extraction

For each included study, we extracted detailed data using a structured form. The following study-level characteristics were collected: study ID, country, study design (randomized or non-randomized trial), and intervention and comparator details. The extracted intervention-related data included the biologic agent used, class of drug, dose and dosage form, and the control treatment. Participant characteristics included total sample size for both intervention and control arms, asthma severity, mean age and standard deviation, gender distribution, and duration of asthma. Additional clinical data included baseline total IgE levels, baseline FEV1 (% predicted), use of inhaled corticosteroids, and duration of follow-up in months.

We also extracted all available efficacy-related outcomes, including clinical response, frequency of asthma exacerbations, pulmonary function metrics (such as FEV1 and FEV1/FVC), quality of life scores, and disease control indices. Similarly, safety outcomes were documented, such as reported adverse events and serious adverse events, including anaphylaxis or adverse events leading to treatment discontinuation.

Because of substantial clinical and methodological heterogeneity across studies including differences in age groups, outcome definitions, study designs, and follow-up durations a quantitative or proportional meta-analysis was not considered appropriate. Therefore, results were synthesized narratively.

### 2.5 Risk of bias assessment

To evaluate the risk of bias, the included studies were assessed. For randomized controlled trials, we used the Cochrane Risk of bias 2.0 (RoB 2.0) tool, which evaluates bias across domains such as the randomization process, deviations from intended interventions, missing outcome data, measurement of outcomes, and selective reporting (14). For non-randomized studies, we used the MINORS (Methodological Index for Non-Randomized Studies) tool, which is specifically designed for the assessment of the methodological quality of non-randomized studies (15). The MINORS tool evaluates 8 criteria for non-comparative studies, including aspects of study aim, inclusion of consecutive patients, data collection, endpoints, follow-up, and statistical analyses. Each item is scored from 0 to 2, with higher total scores indicating better methodological quality.

## 3 Results

### 3.1 Search results and study selection

**Figure 1:**
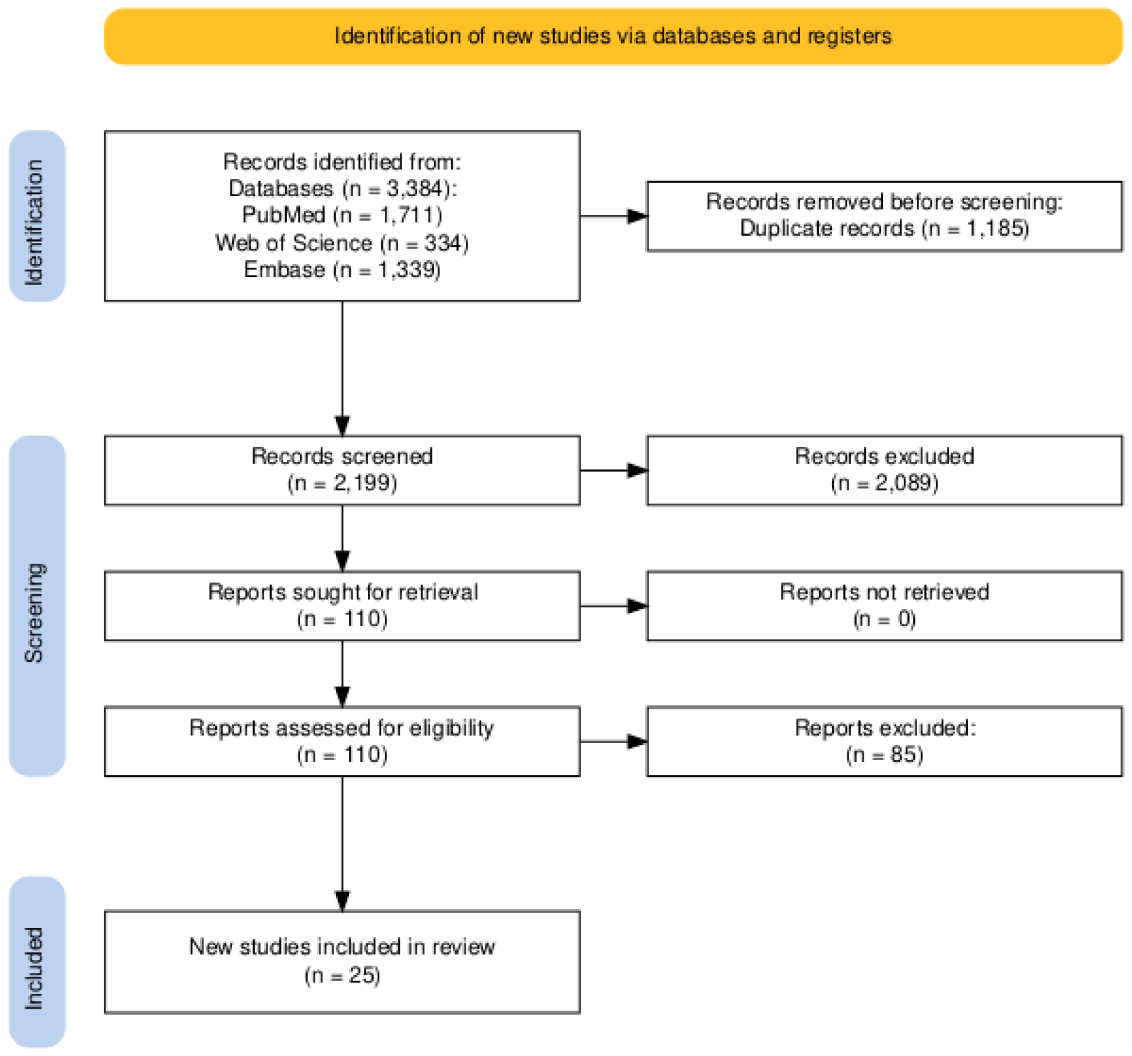
PRISMA flow diagram illustrating the study identification, screening, and selection process.

After thoroughly searching the literature, 3,384 records were identified from various databases, including PubMed, Embase, and Web of Science. After eliminating 1,185 duplicates, the remaining 2,199 records underwent title and abstract screening through Rayyan software.^1^ Our inclusion criteria allowed 110 articles to be identified after carefully screening titles and abstracts. Following a thorough screening of full-text articles, we eliminated 85 records that did not meet specific criteria, resulting in a conclusive selection of 25 studies (16–40) that were included for qualitative synthesis. Furthermore, the reference lists of these research studies were thoroughly scrutinized to identify any relevant publications that might have been missed initially. Figure 1 illustrates the search methodology and PRISMA flow diagram.

### 3.2 Characteristics of included studies

Twenty-five studies were identified and included in systematic review. These studies encompassed major multicentric clinical trials conducted in various regions worldwide including United states, Europe and Asia which were published over different years between 2001 and 2024. While all studies were randomized clinical trials and their extension in addition to non-randomized open label studies in addition to single arm trials, they targeted distinct patient populations, including children and adolescents diagnosed with mainly with moderate to severe asthma with at least 1 year duration and receiving different biologic therapy (Omalizumab, Mepolizumab Dupilumab, Benralizumab, and Lebrikizumab) of different doses. All studies compared biologic therapy to a placebo mainly or steroid therapy. Tables 1, 2 provide detailed summary of the included studies and study populations. Baseline characteristics of participants are shown in Table 3.

**Table 1:** Summary of included study.

Study ID	Location	Study design	Study interventions		Class of drug and doses		Sample size		Follow up
							Biologic	Control	
Bacharier 2023 (41)	Multinational (USA, Australia, France; UK, China)	RCT	Dupilumab	Placebo	Anti-IL-4Rα monoclonal antibody	100 mg (<30 kg) or 200 mg (>30 kg) subcutaneously every 2 weeks	236	114	12 months
Bacharier 2024 (17, 18)	Multinational (Argentina, Australia, Brazil, Canada, Chile, Colombia, Hungary, Italy, Lithuania, Mexico, Poland, Russia, South Africa, Spain, Turkey, Ukraine, USA)	open-label extension study	Dupilumab	Placebo	Anti-IL-4Rα monoclonal antibody	00 mg SC q2w for ≤30 kg, 200 mg SC q2w for >30 kg;	240	125	12 months
Bacharier 2021 (16)	Multicentric in USA, Mexico, Brazil, Argentina, Hungary, Australia, Russia, Poland, South Africa, Turkey, Spain, Ukraine and Romania	RCT VOYAGE (NCT02948959)	Dupilumab	Placebo	IL-4 receptor alpha monoclonal antibody	Subcutaneous injection of dupilumab (at a dose of 100 mg for those weighing ≤30 kg and 200 mg for those weighing >30 kg) every 2 weeks	236	114	52 weeks
							175	84	
Maspero 2024 (33)	Multicentric in USA, Mexico, Brazil, Argentina, Hungary, Australia, Russia, Poland, South Africa, Turkey, Spain, Ukraine and Romania	RCT VOYAGE (NCT02948959)	Dupilumab	Placebo	IL-4 receptor alpha monoclonal antibody	Subcutaneous injection of dupilumab (at a dose of 100 mg for those weighing ≤30 kg and 200 mg for those weighing >30 kg) every 2 weeks	102	50	52 weeks
							131	64	
Phipatanakul 2024 (35)	Multicentric in USA and European countries	Post hoc analysis (open label extension) of VOYAGE (NCT02948959) and EXCURSION (NCT03560466) trials	Dupilumab/Dupilumab	Placebo/Dupilumab	IL-4 receptor alpha monoclonal antibody	Dupilumab 100 mg/2 w (≤30 kg), or 200 mg/2 w (>30 kg) in VOYAGE then entered EXCURSION and received subcutaneous add-on dupilumab 200 mg every 2 weeks or 300 mg every 4 weeks	158	85	52 weeks (VOYAGE) + 52 weeks (EXCURSION) (104 w)
Fiocchi 2023 (24)	Multinational (USA, Australia, France; UK, China)	RCT	Dupilumab	Placebo	Anti-IL-4Rα monoclonal antibody	100 mg or 200 mg SC every 2 weeks	236	114	12 months
Berger 2003 (19)	USA	RCT	Omalizumab	Placebo	Anti-IgE monoclonal antibody (omalizumab)	Subcutaneous omalizumab 150 or 300 mg every 4 weeks or 225, 300, or 375 mg every 2 weeks, based on weight and IgE level	225	109	12 months
Bozek 2024 (22)	Poland	RCT	Omalizumab	Placebo	Anti-IgE monoclonal antibody (omalizumab)	150 mg subcutaneously every 4 weeks for 24 months	17	21	24 months
Chen 2023 (21)	China	RCT	Omalizumab + Budesonide/Formoterol	Budesonide/Formoterol	Anti-IgE monoclonal antibody	150–600 mg subcutaneous injection every 14–28 days	44	44	4 months
Cruz 2007 (21)	Brazil	RCT	Omalizumab	Placebo	Anti-IgE monoclonal antibody (omalizumab)	150 or 300 mg q4w, or 225–375 mg q2w, SC injection	68	69	15 months
Hendeles 2014 (27)	USA	RCT	Omalizumab	Placebo	Anti-IgE monoclonal antibody (omalizumab)	300–375 mg SC every 2 or 4 weeks	15	15	4 months
Lanier 2003 (25)	USA	RCT	Omalizumab	Placebo	Anti-IgE monoclonal antibody (omalizumab)	≥0.016 mg/kg/IgE IU/mL SC every 4 weeks	245	215	6 months
Lanier 2009 (26)	USA	RCT	Omalizumab	Placebo	Anti-IgE monoclonal antibody (omalizumab)	75–375 mg subcutaneous injection every 2 or 4 weeks	421	206	12 months
Milgrom 2001 (32)	Multicentric in USA	RCT	Omalizumab	Placebo	Monoclonal Antibodies – Anti-IgE	Subcutaneous injection of omalizumab every 2 or 4 weeks (at least 0.016 mg/kg/IgE [IU/mL] per 4 weeks) for 28 weeks	225	109	7 months
Lemanske 2002 (40)	Multicentric in USA	RCT	Omalizumab	Placebo	Monoclonal Antibodies – Anti-IgE	Subcutaneous injection of omalizumab every 2 or 4 weeks (at least 0.016 mg/kg/IgE [IU/mL] per 4 weeks) for 28 weeks	225	109	7 months
Silkoff 2004 (31)	Multicentric in USA	RCT with open label extension	Omalizumab	Placebo	Monoclonal Antibodies – Anti-IgE	Subcutaneous injection of omalizumab every 2 or 4 weeks (at least 0.016 mg/kg/IgE [IU/mL] per 4 weeks) for 28 weeks	18	11	28 weeks blinded trial + 24 weeks open extension (52 weeks)
Teach 2015 (38)	Multicentric in USA	RCT	Omalizumab	Placebo	Monoclonal Antibodies – Anti-IgE	Subcutaneous injection of omalizumab every 2 or 4 weeks (at least 0.016 mg/kg/IgE [IU/mL] per 4 weeks) for 28 weeks	225	109	3 months
	Multicentric in USA	RCT with open label extension	Omalizumab	Placebo	Monoclonal Antibodies – Anti-IgE	Subcutaneous injection of omalizumab every 2 or 4 weeks (at least 0.016 mg/kg/IgE [IU/mL] per 4 weeks) for 28 weeks	18	11	
Odajima 2015 (34)	Multicentric at 17 centers in Japan	Open label single arm trial	Omalizumab	NA	Monoclonal Antibodies – Anti-IgE	Subcutaneous injection of Omalizumab 75–375 mg every 2 or 4 weeks according to weight and serum IgE	38		12 months
Zhu 2023 (36)	Multicentric in USA	RCT	Omalizumab	Placebo	Monoclonal Antibodies – Anti-IgE	Subcutaneous injection of Omalizumab 75–375 mg every 2 or 4 weeks according to weight and serum IgE for 52 weeks	351	184	-
Zeitlin 2018 (37)	Multicentric at 30 centres in USA	RCT (NCT02814643)	Benralizumab	Placebo	IL-5 receptor alpha monoclonal antibody	Subcutaneous injection of 30 mg of Benralizumab every 4 weeks (three doses)	51	52	5 months
Busse 2021 (20)	USA, Canada, Sweden	Phase III extension study	Benralizumab Q4W and Q8W	Placebo	Anti–IL-5 receptor α monoclonal antibody	30 mg subcutaneously, Administered every 4 weeks (Q4W) or every 8 weeks (Q8W)	46	40	27 months
Szefler 2022 (30)	Multicentric in USA, Mexico, Brazil, Argentina, Japan, Korea, UK, Germany, France and other European countries	RCT (NCT01875003)	Lebrikizumab	Placebo	Anti-IL-13 monoclonal antibody	Subcutaneous injection of lebrikizumab 125 mg, or lebrikizumab 37.5 mg every 4 weeksfor 52 weeks	37.5 mg (G1) = 113, 125 mg (G2) = 116	117	12 months + 12 months extension + 20 weeks safety follow up (124 weeks)
Gupta 2019 (28)	Japan, Poland, UK, United States	Open-label, Phase 2 study	Mepolizumab	non	Anti–IL-5 monoclonal antibody	“Mepolizumab 40 mg SC every 4 weeks for children <40 kg (n = 26)	36	-	3 months
Jackson 2022 (29)	USA	RCT	Mepolizumab	Placebo	Anti-IL-5 monoclonal antibody	“Age 6–11 years: 40 mg SC every 4 weeks Age 12–17 years: 100 mg SC every 4 weeks”	146	144	12 months
Wilson 2024 (39)	Multicentric at 9 centers in Japan	Substudy of MUPPITS2 RCT (NCT03292588)	Mepolizumab	Placebo	IL-5 receptor monoclonal antibody	Subcutaneous injection of mepolizumab every 4 weeks for 52 weeks (6–11 years: 40 mg; 12–17 years: 100 mg)	22	31	52 weeks

**Table 2:** Study population.

Study ID	Population	Severity of asthma
Bacharier 2023 (41)	Children aged 6–11 years with moderate-to-severe asthma and type 2 inflammation	Moderate-to-severe, uncontrolled
Berger 2003 (19)	Children aged 6–12 years with moderate-to-severe allergic asthma controlled with ICS	Moderate: 90.7% Severe: 9.3%
Bozek 2024 (22)	Patients >16 years with mild to moderate asthma	Mild to moderate asthma
Busse 2021 (20)	Adolescents (aged 13–18) with severe eosinophilic asthma who completed SIROCCO or CALIMA trials	Severe eosinophilic asthma
Bacharier 2024 (17, 18)	Children aged 6–11 with moderate-to-severe asthma, completed VOYAGE study	Moderate-to-severe
Chen 2023 (21)	Children aged 6–11 years with moderate to severe allergic asthma	Moderate and severe
Cruz 2007 (21)	Adolescents and young adults with allergic asthma and/or perennial allergic rhinitis at high risk of geohelminth infection	Mild to moderate asthma
Fiocchi 2023 (24)	Children aged 6–11 years with uncontrolled moderate-to-severe type 2 asthma	Moderate-to-severe
Gupta 2019 (28)	Children aged 6–11 with severe eosinophilic asthma, ≥2 exacerbations in prior year	Severe eosinophilic asthma
Hendeles 2014 (27)	Children and young adults (ages 6–26) with persistent allergic asthma and <50% ICS adherence	Persistent allergic asthma with poor control
Jackson 2022 (29)	Urban children and adolescents (6–17 years) with exacerbation-prone asthma, living in low-income census tracts, with ≥2 exacerbations in the prior year and blood eosinophils ≥150 cells/mm^3^	Exacerbation-prone eosinophilic asthma
Lanier 2003 (25)	Patients with severe allergic asthma uncontrolled on ICS (12–73)	Severe persistent asthma
Lanier 2009 (26)	Children aged 6 to <12 years with moderate-to-severe persistent allergic asthma	Moderate-to-severe persistent allergic (IgE-mediated) asthma
Milgrom 2001 (32)	Children aged 6 to 12 years of both sexes, with moderate to severe allergic asthma for at least 1 year and positive skin-prick test to certain antigens	Moderate to severe asthma on ICS
Lemanske 2002 (40)	Children aged 6 to 12 years, with allergic asthma for at least 1 year and positive skin-prick test to at least 1 common aeroallergen	Moderate to severe well-controlled asthma with ICS
Silkoff 2004 (31)	Children aged 6 to 12 years, with allergic asthma for at least 1 year and positive skin-prick test to at least 1 common aeroallergen	Moderate to severe well-controlled asthma with ICS
Teach 2015 (38)	Children and adolescents aged 6 to 17 years, with asthma for more than 1 year,1 or more asthma exacerbations requiring systemic corticosteroids or hospitalization within the prior 19 months, a positive skin test response at least one allergen	Mild, moderate and severe asthma
Odajima 2015 (34)	Children aged 6–15 years with a diagnosis of severe persistent allergic asthma according to the Japanese pediatric guideline uncontrolled with ICS	Severe uncontrolled asthma
Zeitlin 2018 (37)	Adolescents and young adult patients aged 12–21 years receiving medium- to high-dosage of ICS	Moderate to severe asthma
Szefler 2022 (30)	Patients aged 12–17 years with uncontrolled asthma diagnosed ≥12 months previously and on at least 6 months of ICS and other asthma medications	Uncontrolled or severe asthma despite standard care
Zhu 2023 (36)	Children aged 6 to 11 years with moderate-to-severe, persistent, inadequately controlled allergic asthma	Moderate to severe inadequately controlled allergic asthma
Bacharier 2021 (16)	Children aged 6 to 11 years with diagnosis of persistent asthma for ≥12 months prior to screening on ICS or other asthma medication for at least 3 months (Type 2 Inflammatory Phenotype)	Moderate-to-severe asthma
	Children aged 6 to 11 years with diagnosis of persistent asthma for ≥12 months prior to screening on ICS or other asthma medication for at least 3 months (≥300 Blood Eosinophils per mm3)	
Maspero 2024 (33)	Children (aged 6–11 years) with uncontrolled moderate to severe asthma on high dose ICS	Moderate-to-severe uncontrolled asthma
	Children (aged 6–11 years) with uncontrolled moderate to severe asthma on medium-dose ICS	
Wilson 2024 (39)	Urban children and adolescents 6–17 years of age with exacerbation-prone asthma and blood eosinophils ≥150 cells/mm3.	Severe eosinophilic asthma
Phipatanakul 2024 (35)	Children aged 6–11 years with moderate-to-severe asthma who completed VOYAGE trial then entered EXCURSION study	Moderate-to-severe asthma

**Table 3:** Baseline characteristics of participants.

Study ID	Age, mean (SD) y		Gender (male), n (%)		Duration pf asthma, mean (SD) years		Total IgE, IU/mL, mean(sd)		Use of ICS, n(%)	
	Biologic	Control	Biologic	Control	Biologic	Control	Biologic	Biologic	Biologic	Control
Bacharier 2023 (41)	8.9 (1.6)	9.0 (1.6)	84 (35.6)	36 (31.6)	NR	NR	530.0 (213.0–1268.0)	397.0 (144.0–862.0)	102 (43.2)	50 (43.9)
Berger 2003 (19)	9.4 (5–12)	NR	158 (70.2)	NR	6.1 (1–12)	NR	348 (20–1,269)	NR	NR	NR
Bozek 2024 (22)	23.1 (5.4)	26.2 (7.1)	9 (52.9%)	10 (47.6%)	5.0 (3.2)	4.8 (2.2)	30–850 IU/mL	30–850 IU/mL	9 (SABA+ICS), 8 (LABA+ICS)	9 (SABA+ICS), 12 (LABA+ICS)
Busse 2021 (20)	15.6 6 1.39	15.5 6 1.63	27	24	NR	NR	NR	NR	NR	NR
Bacharier 2024 (17, 18)	8.9 (1.6)	8.9 (1.6)	136 (65.1%)	72 (67.9%)	NR	NR	942.5 (1168.6)	767.3 (1102.2)	91 (43.5%)	49 (46.2%)
Chen 2023 (21)	8.52 (1.12)	8.39 (1.03)	30 (68.18%)	28 (63.64%)	3.34 (0.48)	3.45 (0.55)	226.53 (21.62)	230.39 (21.26)	NR	NR
Cruz 2007 (21)	15 (12–29)	16 (12–30)	33 (48.5)	25 (36.2)	11	12	403 (39–1,100)	397 (50–231)	NR	NR
Fiocchi 2023 (24)	8.9 (±1.6)	9.0 (±1.6)	152 (64.4%)	78 (68.4%)	NR	NR	NR	NR	102 (43.2%)	50 (43.9%)
Gupta 2019 (28)	8.6 (1.9)		25 (69%)		NR		348 (SD log 1.36)		36 (100%)	
Hendeles 2014 (27)	16.4 (5.5)	16.4 (5.5)	7 (41.2%)	7 (41.2%)	NR	NR	427 (275)	427 (275)	15 (100%)	15 (100%)
Jackson 2022 (29)	10.0 (9.0–13.0)	11.0 (9.0–13.0)	76 (52%)	91 (62%)	NR	NR	NR	NR	NR	NR
Lanier 2003 (25)	39.6 (12–73)	38.6 (12–74)	95 (38.8)	96 (44.7)	20.4 (2–61)	22.6 (2–60)	173.4 (21–860)	186.2 (21–702)	NR	NR
Lanier 2009 (26)	8.7 (1.7)	8.4 (1.7)	287 (68.2)	138 (66.7)	NR	NR	476.0 (339.3)	456.9 (335.8)	367 (87.2)	182 (87.9)
Milgrom 2001 (32)	9.4 (1.17)	9.5 (1)	158 (70.2)	73 (67.0)	6.1 (1.83)	6.1 (1.83)	348 (208.17)	323 (197.17)	225 (100)	109 (100)
Lemanske 2002 (40)	9.4 (1.17)	9.5 (1)	158 (70.2)	73 (67.0)	6.1 (1.83)	6.1 (1.83)	348 (208.17)	323 (197.17)	225 (100)	109 (100)
Silkoff 2004 (31)	8.8 (1.8)	10.8 (0.8)	15 (38.33)	5 (45.46)	more than 1 y		315.6 (279.7)	301.7 (213.0)	18 (100)	11 (100)
Teach 2015 (38)	10.4 (3.11)	9.84 (2.70)	78 (64.5)	70 (53.8)	7.62 (3.80)	6.87 (3.28)				
	10.3 (2.99)	10.1 (3.06)	174 (67.2)	59 (66.3)	7.72 (3.56)	7.24 (3.56)				
Odajima 2015 (34)	10.7 (2.46)		23 (60.5)		8.4 (3.05)		335.5 (254.1)		38 (100)	
Zeitlin 2018 (37)	16 (2.25)	16 (2.25)	30 (58.8)	31 (59.6)					51 (100)	52 (100)
Szefler 2022(30)	G1: 14.2 (1.5), G2: 14.2 (1.6)	14.1 (1.7)	G1: 70 (62%), G2: 57 (49%)	68 (58%)	G1 and G2: 11 (2.67)	9 (2.67)	G1: 361 (150.17), G2: 368 (125.17)	358 (138)	G1: 113 (100), G2: 116 (100)	117 (100)
Zhu 2023 (36)	8.6 (1.69)						950 (810)		535 (100)	
Bacharier 2021 (16)	8.9 (1.6)	9.0 (1.6)	152 (64.4)	78 (68.4)	more than 1 y		670.33 (786.9)	467.67 (539.11)	236 (100)	114 (100)
	8.9 (1.6)	9.0 (1.5)	116 (66.3)	58 (69.0)			800.33 (851.4)	623.33 (668.3)	175 (100)	84 (100)
Maspero 2024 (33)	2.9 (2.6)	3.3 (2.5)	64 (622.7)	33 (66)	more than 1 y		596.7 (628.8)	444 (598.4)	102 (100)	50 (100)
	9.0 (1.6)	8.9 (1.7)	86 (65.6)	45 (70.3)			714.3 (905.4)	495 (561.4)	131 (100)	64 (100)
Wilson 2024 (39)	12.5 (1.9)	12.1 (1.9)	7 (32)	23 (74)			612.3 (685.5)			
Phipatanakul 2024 (35)					more than 1 y				158 (100)	85 (100)

### 3.3 Risk of bias

**Figure 2:**
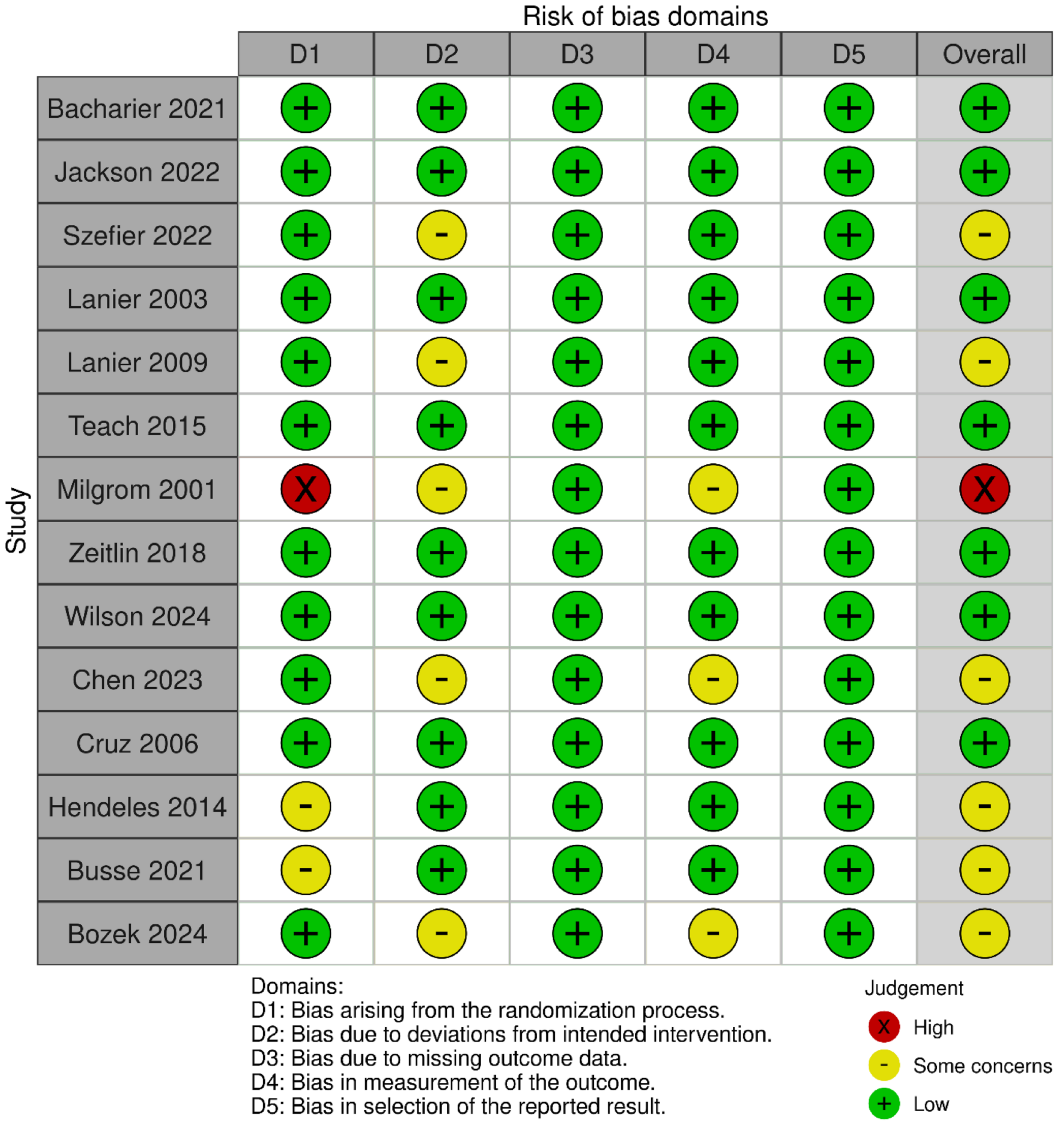
Risk of bias assessment of RCTs using Rob-2.

The ROB2 tool was used to perform quality assessment of RCTs included in the review. The risk of bias evaluation revealed that clinical trials (16, 21, 25, 29, 37–39) had overall assessment as low risk, with a low-risk judgment in all five domains. Additionally, other clinical trial (20, 22, 23, 26, 27, 30) raised some concerns regarding the overall assessment, primarily due to potential biases in randomization, deviation in interventions or blinding process while Milgrom et al. (32) had overall high risk of bias due to potential bias in randomization process as shown in Figure 2. Furthermore, MINORS assessment for single arm and interventional non comparative studies showed moderate quality in studies (28, 34, 35) (Table 4).

**Table 4:** MINORS scale for included single arm and non-randomized interventional studies.

Study	I	II	III	IV	V	VI	VII	VIII	Total
Odajima 2015 (34)	2	0	1	2	0	2	1	0	8
Gupta et al. 2019 (28)	1	2	1	2	1	2	0	0	9
Phipatanakul 2024 (35)	2	1	1	2	0	1	2	0	9

Numbers I-VIII in heading domains include: I, a clearly stated aim; II, inclusion of consecutive patients; III, prospective collection of data; IV, endpoints appropriate to the aim of the study; V, unbiased assessment of the study endpoint; VI, follow-up period appropriate to the aim of the study; VII, loss of follow up less than 5%; VIII, prospective calculation of the study size.

### 3.4 Clinical and efficacy outcomes

#### 3.4.1 Asthma exacerbations

Dupilumab exhibited significant improvements in asthma exacerbation rate across many studies. In VOYAGE clinical trial conducted on children aged 6 to 11 years with diagnosis of persistent eosinophilic moderate to severe asthma for at least 1 year, dupilumab reduced severe exacerbation rates by 59.3% compared to placebo with annualized exacerbation rate (AER) of 0.31 vs. 0.75/year for placebo group in children with phenotype 2 asthma. In patients with high blood eosinophilia (≥300 eosinophils/μL), the reduction of exacerbations was 64.7% in dupilumab group with AER of 0.24 versus 0.67/year in control group. All results were statistically significant (*p* < 0.001) favoring biologic therapy in both subgroups. Additionally, over 52 weeks, more patients on dupilumab experienced no asthma exacerbations (77.1%) compared to 59.6% of patients in control group in phenotype 2 asthma patients, while it was 79.0% vs. 58.3%, respectively, in patients with high eosinophilia subgroup. Regarding time to first exacerbation, dupilumab delayed first severe exacerbation with hazard ration (HR): 0.44 versus 0.38 in control group.

Children who completed 52-week VOYAGE trial entered EXCURSION an open-label extension study by Bacharier et al. (17, 18) in which both dupilumab and placebo groups received add-on subcutaneous dupilumab 100 mg or 200 mg every 2 weeks according to body weight for additional 52 weeks. During this open label study, dupilumab persistently reduced the rate of severe asthma attacks. The unadjusted AER was even lower than the rate observed in the dupilumab group during VOYAGE study, indicating a sustained and clinically significant benefit with subsequent reduced use of systemic corticosteroids AER: 0.118 (dupilumab/dupilumab) vs. 0.124 (placebo/dupilumab). Moreover, 91% of patients remained exacerbation-free during study.

In a post-hoc analysis by Maspero et al., assessing stratified patients in VOYAGE trial into subgroups by ICS controller requirement at baseline (medium-dose ICS plus second controller or high-dose ICS with or without a second controller) to evaluate the effects of ICS doses on clinical response and asthma exacerbations. The study reported that dupilumab significantly reduced severe exacerbations compared to placebo by 63% in those on high-dose ICS and 59% in those on medium-dose ICS in children with type 2 asthma indicating higher efficacy of dupilumab in ICS users.

Similarly, Milgrom et al., reported that omalizumab had significantly fewer asthma exacerbations (18.2% vs. 38.5%) in placebo, fewer episodes per patient (0.42 vs. 2.72), and no hospitalizations, unlike 5 asthma exacerbations requiring hospitalization in placebo group during steroid-reduction phase. While during stable steroid phase, omalizumab showed fewer asthma exacerbations compared to placebo but it did not reach statistical significance. Moreover, Teach et al., reported the superiority of omalizumab regarding risk of having at least one asthma exacerbation over 90 days compared to placebo (11.3% vs. 21.0%; OR 0.48). However, no significant difference was seen between omalizumab and ICS boost in participants at steps 2–4 of asthma treatment (8.4% vs. 11.1%; OR 0.73). Most exacerbations (89%) were linked to respiratory viruses, mainly rhinovirus (81%). Additionally, rhinoviruses were more common during exacerbations (57% vs. 36%; OR 2.32). Omalizumab showed a trend toward fewer virus-related exacerbations (OR 0.52). Furthermore, Odajima et al., reported lower AER over the 24-week treatment period compared with baseline (*p* < 0.001) especially among patients treated with high-dose ICS. Notably, fewer exacerbations were observed in omalizumab [0.60 vs. 0.83 per patient (*p* = 0.023)] in addition to fewer patients with ≥1 exacerbation: (31.8% vs. 42.8%, *p* = 0.015) as reported by Lanier et al.

Mepolizumab also showed significant reduction in exacerbation attacks as reported by Jackson et al. (29) and Gupta et al. (28). However, Wilson et al. (39) reported that higher levels of activated CD62Lint and CD62Lhi eosinophils in sputum were more likely to experience exacerbations, despite treatment compared to other eosinophils (*p* = 0.04).

As reported by Szefler et al. (30), lebrikizumab significantly reduced asthma exacerbation rates and increased time to first exacerbation especially those with high eosinophil at both 125 mg and 37.5 mg doses compared to placebo over 52 weeks. Regarding benralizumab, Busse et al. (20) reported lower exacerbation rates in benralizumab with 75% of patients were free of attacks unlike Zeitlin et al. (37) which reported similar exacerbation rate between benralizumab and placebo during study.

#### 3.4.2 Clinical response and control of asthma symptoms

Omalizumab 150 mg every 4 weeks showed significant reductions in total asthma symptom score (TASS) and total medication score with good asthma control over the 24-month period as reported by Bozek et al. Additionally, in a multicentric trial by Milgrom et al. Investigators and patient global evaluation of clinical efficacy favored the omalizumab group over the placebo group (both *p* < 0.001) with excellent response for 31.5% of the omalizumab group versus 16.3% in placebo. Similarly, Lanier et al. found that 79% of omalizumab-treated patients achieved “excellent” or “good” response versus 56% in the placebo group evaluated by investigators. While, it was 80% vs. 72%, respectively on patient scale (both *p* < 0.001) over 52 weeks Moreover, patients treated with omalizumab demonstrated sustained asthma control during the 24-week extension phase while receiving lower doses of inhaled corticosteroids.

Furthermore, Chen et al., reported improvement in childhood asthma-control test (C-ACT) in omalizumab plus budesonide/formoterol group compared to control (budesonide/formoterol) with higher scores in favor of omalizumab with significant overall clinical response rate (84.09% (omalizumab) vs. 61.36% (control), *p* < 0.05). In PROSE trial, Omalizumab significantly reduced daily asthma symptoms versus placebo, but showed no significant difference compared to ICS boost over 12 weeks, While Odajima et al., reported that omalizumab significantly improved total asthma symptom after 24 weeks compared to baseline.

In multicentric VOYAGE trial (24), dupilumab-treated patients had higher percentage of achieving improvement with 0.5 points or greater on ACQ-7 compared to control group over 52 weeks (86% vs. 75%, *p* = 0.0051). On the other hand, Jackson et al., showed that patients treated with mepolizumab demonstrated sustained asthma control compared to control similar to Gupta et al., who found that 48% of mepolizumab-treated children achieved ≥0.5-point improvement in ACQ-7 score with notable improvement on C-ACT by week 12. In contrast, both benralizumab and placebo groups showed modest improvement in ACQ-6 scores by week 4, maintained through week 12 with no statistically significant difference was seen in the percentage of poorly controlled patients at week 12 (35.3% vs. 36.5%) respectively as reported by Zeitlin et al. (37) and Busse et al. (20).

#### 3.4.3 Quality of life outcomes

Lemanske et al. and Odajima et al., showed that omalizumab-treated patients had statistically significantly greater improvements in all life quality domains (activities, emotions, symptoms and overall score) on PAQLQ scale from baseline. Additionally, more patients on omalizumab showed a significant clinical improvement in asthma-related quality of life, especially in daily activities and overall scores compared to placebo group. However, Lanier et al. reported light improvement in PAQLQ score in omalizumab group but with no statistical significance.

In the VOYAGE trial, dupilumab showed statistically significant improvement in QoL of children evaluated by PAQLQ-IA (1.58 vs. 1.29) and caregivers’ quality of life on PACQLQ (1.75 vs. 1.29) over 52 weeks compared to control group. Similarly, benralizumab showed greater improvement in AQLQ over 108 weeks especially in patients previously treated with benralizumab (20). In contrast, no significant change in quality of life by the AQLQ was observed with either lebrikizumab doses compared to placebo as reported by Szefler et al. (30).

#### 3.4.4 Pulmonary function

In the VOYAGE trial, dupilumab improved ppFEV1 more than placebo in type 2 asthma (10.5% vs. 5.3%; difference: +5.2%, *p* < 0.001) by week 12 with sustained benefits up to week 52. In addition, baseline values increased from 78 to 87.8% with dupilumab and to 83.2% with placebo. Similar results were seen in patients with high eosinophilic asthma. In accordance with ppFEV1 results, dupilumab showed significant improvement in FEV1, FEF25-75%, FEV1/FVC ratio compared to placebo (*p* < 0.001 to 0.01) as reported by Bacharier et al. (41). In *post hoc* analysis for VOYAGE trial, Maspero et al. reported that the greatest improvements in ppFEV1 were seen in those on medium-dose ICS. In children with allergic asthma on high-dose ICS, the improvement was only significant at week 4. However, there were no significant differences were found between ICS subgroups at week 52.

Regarding omalizumab, Milgrom et al., reported little changes in spirometry measurements with minimal differences between the treatment groups similar to Berger et al., and Hendeles et al., who found that FEV1 had no significant changes during study period. On other hand, Lanier et al., found that omalizumab-treated patients showed statistically significant improvements in FEV1 at weeks 32, 36, 40, and 44 compared to placebo. These findings were consistent with Chen et al., who reported significant improvements in FEV1, PEF, FVC in omalizumab-treated patients compared to budesonide/formoterol group (*p* < 0.05).

Jackson et al., showed that mepolizumab had no significant difference in ppFEV1 or FEV1/FVC compared to control while lebrikizumab 37.5 mg showed a placebo-corrected increase in pre-bronchodilator FEV₁ of 198 mL, while the 125 mg dose showed a smaller, non-significant increase of 53 mL at week 52 as reported by Szefler et al. (30).

### 3.5 Safety outcomes

#### 3.5.1 Common adverse events

Milgrom et al. and Lanier et al., reported comparable adverse events between omalizumab and control groups which were mild to moderate severity. Treatment-emergent adverse events (TEAEs) and urticaria were higher in the omalizumab 0.016 mg/kg/IgE/4 W (6.2% vs. 0.9%) and (4% vs. 0.9%) respectively. Similarly, PROSE trial showed similar rates of non-significant adverse events in the omalizumab (75 mg -375 mg/2-4 W) (54.5%), placebo (54.8%) and ICS group (43.5%). The most common adverse events related to omalizumab and benralizumab were nasopharyngitis, upper respiratory tract infection (URTI) gastroenteritis and headache and events were of mild to moderate severity as reported by Odajima et al., Berger et al. and Zeitlin et al. A study by Cruz et al., reported comparable non serious adverse events especially, skin reactions (44.1% omalizumab vs. 21.7% placebo) and geohelminth infections (50% vs. 41%) in addition to more mild elevations in liver enzymes related to omalizumab. Chen et al., reported slightly higher adverse events in omalizumab (150–600 mg/2-4 W) plus budesonide/formoterol compared to budesonide/formoterol alone (9.09% vs. 4.55%), however, it did not reach statistical significance (*p* = 0.398).

In VOYAGE trial, adverse event rates were comparable between dupilumab and placebo (83% vs. 79.9%) and URTIs, in particular viral infections, were more common with dupilumab (12.2% vs. 9.7%). Additionally, TEAEs occurred in 63% for dupilumab compared to 71–73% in placebo group over 52 weeks. These comparable rates of non-serious TEAEs continued for additional 52-week EXCURSION extension study which reported rate of 84–85% in dupilumab patients who continued dupilumab and 77–79% in placebo patients who shifted to dupilumab 200 mg/2 W.

Regarding mepolizumab (40-100 mg/4 W), injection-site reactions and headache were common during studies with 67% treatment related events as reported by Gupta et al. and Jackson et al. In a multicentric RCT by Szefler et al., lebrikizumab-treated patients (both 125 mg and 37.5 mg) experienced comparable TEAEs compared to placebo (68% vs. 62%).

#### 3.5.2 Serious adverse events (SAE)

Lanier et al. (26) reported less frequent SAE in the omalizumab group compared to placebo (4% vs. 8%, *p* < 0.05) with one anaphylaxis case per group. Similarly, SAE was (1.2% vs. 1.4%) and (1.5% vs. 2.9%) in Lanier et al. (25) and Cruz et al. (21) respectively. Additionally, Berger et al. reported 4 cases with SAE omalizumab-treated patients but were not related to treatment while Bozek et al. Milgrom et al. and Hendeles et al., reported that omalizumab had no SAE or events leading to study discontinuation. On other hand, two SAEs were reported during PROSE trial including one patient in control group with 7th nerve palsy and one in ICS group had anaphylaxis. No deaths or non-asthma hospitalizations occurred with omalizumab. These findings indicate safe profile favoring omalizumab. However, two cases with SAE were related to omalizumab but resolved with treatment and no reported anaphylaxis or platelets abnormalities as reported by Odajima et al. (34).

In VOYAGE, SAE and discontinuations were 4.8% in dupilumab versus 4.5% in placebo. Eosinophilia occurred more with dupilumab (5.9% vs. 0.7%), and was mild except for one case was serious. Moreover, no permanent discontinuation or deaths in dupilumab or placebo groups while hospitalization rate was 1.5% in dupilumab versus no cases in placebo. In EXCURSION, there was no SAE led to study discontinuation with rare SAE (6%) among patients who continued or shifted to dupilumab.

Among patients treated with lebrikizumab of 125 mg and 37.5 mg doses, SAEs were rare 3% and similar to placebo group (3%) with discontinuation due to adverse events (2% vs. 1%), however, no deaths were reported among both groups. For mepolizumab, 6 children (17%) had SAEs and 2 children discontinued study due severe asthma exacerbation and multiple intolerable symptoms as reported by Gupta et al., while in a study by Jackson et al., 3 cases received mepolizumab vs. 2 patients in placebo group experienced anaphylaxis and one death occurred in mepolizumab group due to severe asthma exacerbation. On the other hand, no frequent serious adverse events (8%) and no deaths were observed with benralizumab as reported by Busse et al., and Zeitlin et al., respectively.

## 4 Discussion

Asthma is one of the most common chronic diseases in children, with approximately 2–10% of children suffer from severe asthma which remains poorly controlled despite maximal standard therapy (42, 43). These children experience frequent symptoms and asthma exacerbations leading to hospitalizations and even risk of mortality. Moreover, dependence on systemic corticosteroids for severe asthma management carries significant short- and long-term risks such as, growth suppression, bone fragility, infections, and even a single oral steroid burst can increase risks of sepsis, pneumonia, and fractures in children (44, 45). Biologic therapies have emerged to fulfill need of steroid-sparing therapies that more directly target and selectively modulate the underlying immunological drivers of severe asthma, offering a phenotype-directed approach beyond traditional inhaled therapy (41).

Although biological agents have been widely used in asthma treatment for nearly 20 years and are backed by extensive evidence of their efficacy and safety, studies focusing on their use in children and adolescents are still limited and not at the same pace as those for adult populations (46). With increasing prevalence of asthma among younger populations, it is crucial to assess how these biological treatments affect clinical aspects in pediatric cases to better guide medical decision-making. Currently, the body of research concerning childhood and adolescent asthma remains notably underdeveloped. This systematic review aims to close this gap of knowledge and by integrating data from 25 studies and reviewing existing literature about different biologic therapy in children and adolescents.

Dupilumab is a fully human monoclonal antibody directed against the IL-4 receptor alpha chain, which is a shared component of the receptors for IL-4 and IL-13. By blocking IL-4Rα, dupilumab simultaneously inhibits IL-4 and IL-13 signaling, thereby shutting down two central drivers of type 2 inflammation resulting in downstream reductions in IgE levels, eosinophil recruitment, and biomarkers like fractional exhaled NO (FeNO) and periostin (41, 47). Notably, dupilumab showed significant reduction of exacerbations in children with moderate to severe asthma with long-term effect sustained over 104 weeks as reported in VOYAGE and EXCURSION studies and this highlighted that efficacy was maintained across different doses of ICS. Based on the VOYAGE trial data, dupilumab led to an 86% improvement in ACQ-7 scores versus 75% in placebo over 52 weeks with prominent improvements as early as Week 12, indicating better control of asthma symptoms. Additionally, significant improvement in QoL in patients and caregivers using PAQLQ-IA and PACQLQ, showing benefit beyond clinical symptom control. The most pronounced improvement in lung function among biologics was seen with dupilumab, which enhanced ppFEV1 significantly from baseline to Week 12 and sustained to Week 52. Improvement extended to other pulmonary markers such as FEV1, FEF25-75%, and FEV1/FVC ratios. Regarding safety outcomes, URTI rates and mild eosinophilia were frequent events in patients receiving dupilumab, but overall rates of adverse events were comparable to placebo with no reported deaths in both VOYAGE and EXCURSION trials.

Omalizumab was the first monoclonal antibody introduced for asthma and is indicated for moderate-to-severe allergic asthma. It binds to the Fc portion of free IgE, preventing IgE from attaching to FcεRI receptors on mast cells, basophils, and dendritic cells (48). Omalizumab reduced exacerbation rates significantly with a drop in hospitalization episodes and lower exacerbation frequency as reported by Milgrom et al. Similarly, Teach et al. reported a significant reduction in virus-induced exacerbations, with rhinovirus being a primary trigger. These findings suggest a dual anti-inflammatory and anti-viral effect of omalizumab. Furthermore, Omalizumab significantly reduced asthma symptom scores over long-term periods, as shown in Bozek et al. and Lanier et al. It also improved patient and investigator-rated global efficacy evaluations and PAQLQ suggesting improvement of asthma symptoms and QoL. On the other hand, omalizumab showed debatable spirometry results as significant FEV1 gains were seen in Lanier et al. and Chen et al., while, minimal to no changes were observed in Milgrom et al. and Hendeles et al. highlighting the inconclusive evidence about lung function improvement. However, omalizumab was associated with URTI, rash, and mild hepatic enzyme elevations. Some skin-related and parasitic infections with rare SAEs and no treatment-related deaths were noted.

Mepolizumab is a humanized monoclonal antibody targeting IL-5, the key cytokine for eosinophil differentiation and survival. It is approved for children over 6 years with severe eosinophilic asthma (41). Mepolizumab, while showing efficacy in many cases, demonstrated varied responses. In studies by Jackson et al. and Gupta et al., mepolizumab was effective in reducing attacks; however, Wilson et al., highlighted a potential for increased exacerbations in patients with activated eosinophil subsets. Additionally, mepolizumab showed modest to non-significant FEV1 improvements indicating minimal effects on pulmonary function. Mepolizumab induced headache and injection site reactions, especially at higher doses were observed. In addition, it was associated with higher SAE and discontinuation rates in children, including one reported death.

Regarding other biologics, lebrikizumab and benralizumab exhibited promising but less consistent results. Szefler et al., found improved time to first exacerbation with lebrikizumab. However, Zeitlin et al., reported no statistical difference between benralizumab and placebo. Furthermore, benralizumab and lebrikizumab showed only modest symptom improvements, with no significant advantage over placebo unlike other biologics. Interestingly, Benralizumab improved QoL only in previously treated patients, while lebrikizumab did not show QoL improvements as reported by Szefler et al. Regarding lung function, benralizumab showed modest to non-significant FEV1 improvements while, lebrikizumab had a modest benefit only with the 37.5 mg dose indicating lack of efficacy of these emerging biologics in pediatric asthma. However, they showed good safety profile associated with non-significant TEAEs compared to placebo with low SAE rates and no deaths.

### 4.1 Previous research

Previous studies have discussed the safety and efficacy associated with biologic therapy, Lin et al. (49) conducted a network meta-analysis that evaluated safety and efficacy of four biologics including dupilumab, omalizumab, lebrikizumab, and mepolizumab among asthmatic children and adolescents. Although they discussed the effects of these agents, their review was restricted to eight clinical trials, thus offering a more limited perspective.

Similarly, Wirthgen et al. (50), conducted a review evaluating the effects of 5 biologics on pediatric children with asthma; however, their study focused on safety outcomes only demonstrating no new safety concerns regarding the use of biologics in pediatric asthma. Additionally, Fenu et al. (51), conducted a meta-analysis for omalizumab in children with asthma which revealed significant reduction of exacerbation with decreased need for steroid treatment, however, it was confined to one biologic therapy and included only 4 trials.

### 4.2 Strengths and limitations

This systematic review highlights the growing body of evidence supporting the efficacy and safety of biologic therapies for pediatric asthma across twenty-five studies of five different biological therapy compared to placebo, ICS or other treatments giving conclusive evidence about efficacy and safety of these drugs. *Post hoc* analyses and extended open label studies raised insights into drug efficacy in specific subgroups, such as those with medium and high doses of ICS, and provide important nuance to treatment response. However, there are some limitations that must be declared: the heterogeneity in the designs of studies, primarily on dose of treatment; furthermore, restricted evidence on emerging biologics including lebrikizumab and benralizumab due to limited clinical trials may impair the generalizability and evidence for these drugs. The inclusion of patients with mild-to-moderate as well as moderate-to-severe asthma may have influenced outcome magnitude, as biologic efficacy is known to vary by disease severity. This review included studies enrolling patients across a spectrum of asthma severity, ranging from mild-to-moderate to severe asthma, as well as adolescents and young adults in addition to children. While this broad inclusion enhances external validity, treatment responses and outcomes may differ across severity strata and age groups, which should be considered when interpreting the findings. The review protocol was not prospectively registered in PROSPERO. Although this decision was made due to concerns regarding premature disclosure of a highly focused and emerging topic, lack of prospective registration may increase the risk of selective reporting and should be considered when interpreting the findings. Moreover, *post hoc* analyses are informative but exploratory in nature and may be subject to potential bias, given that they were not pre-defined endpoints. Finally, although benefits are observed from these trials over short- to medium-term periods, further studies are required to establish the long-term effects of different biologic therapy to determine the best biological option for pediatric asthma.

## 5 Conclusion

In conclusion, dupilumab and omalizumab demonstrated substantial benefits in reducing exacerbations, improving symptom control, and enhancing quality of life. While mepolizumab showed moderate efficacy, its impact on lung function and adverse event rates warrants caution. The clinical outcomes for lebrikizumab and benralizumab were modest and less consistent, likely due to limited data. Notably, dupilumab provided the most consistent and sustained improvements across multiple outcomes, including lung function and patient-reported outcomes for life quality. This review may serve as a reference for informed clinical decision-making and future investigation.

## Data Availability

The original contributions presented in the study are included in the article/Supplementary material, further inquiries can be directed to the corresponding author.

## Footnotes

1
https://new.rayyan.ai/reviews
