# Supplementary material for: Comparative efficacy and safety of biologic therapies in pediatric asthma: a comprehensive systematic review
Source: Front Med (Lausanne). 2026 Mar 18;13:1722577. doi: 10.3389/fmed.2026.1722577 (PMC13038997; doi:10.3389/fmed.2026.1722577)
Supplement: Supplementary file 1 [file Table_1.docx]

**Supplementary file**

**Search strategy according each database**

**PubMed and Web of science (WOS)**

[1] "Asthma"[Mesh] OR Asthmas OR "Bronchial Asthma" OR "Allergic Asthma" OR " Extrinsic asthma" OR "Induced Asthma" OR "Neutrophilic Asthma" OR asthma =232732

[2] "Antibodies, Monoclonal, Humanized"[Mesh] OR "Humanized Antibodies" OR "Monoclonal antibodies" =329660

[3] "Omalizumab"[Mesh] OR Xolair OR "benralizumab" [Supplementary Concept] OR MEDI-563 OR MEDI 563 Z OR Fasenra OR BIW-8405 OR "mepolizumab" [Supplementary Concept] OR SB-240563 OR SB240563 OR Nucala OR Bosatria OR "dupilumab" [Supplementary Concept] OR SAR231893 OR SAR-231893 OR REGN668 OR REGN-668 OR Dupixent OR "tezepelumab" [Supplementary Concept] OR tezspire OR AMG-157 OR MEDI-19929 OR MEDI9929 OR MEDI-9929 OR "lebrikizumab" [Supplementary Concept] OR ebglyss OR RG-3637 OR PRO301444 OR MILR-1444A OR MILR1444A OR "reslizumab" [Supplementary Concept] OR CEP38072 OR CEP-38072 OR DCP 835 OR DCP-835 OR SCH55700 OR Cinqair OR SCH-55700 OR RHUMAB-E25 OR lebrikizumab OR TNX-650= 9977

[4] [2] OR [3] =333545

[5] ("Adolescent"[Mesh] OR Adolescents OR Adolescence OR Youth OR Teens OR Teen OR children OR Teenager OR Teenagers OR Youths) OR Pediatric =5,059,854

[1] AND [4] AND [5] = 1711

**WOS = 334**

**EMBASE**

1 Asthma.mp. or Asthma, Exercise-Induced/ or Asthma/ =211844

2 (Asthmas or "Bronchial Asthma" or "Allergic Asthma" or " Extrinsic asthma" or "Induced
 Asthma" or "Neutrophilic Asthma" or asthma).mp. [mp=title, book title, abstract, original title,
 name of substance word, subject heading word, floating sub-heading word, keyword heading
 word, organism supplementary concept word, protocol supplementary concept word, rare
 disease supplementary concept word, unique identifier, synonyms, population supplementary
 concept word, anatomy supplementary concept word] =211853

3 1 or 2 211853

4 Antibodies, Monoclonal, Humanized.mp. or Antibodies, Monoclonal, Humanized/ =60791

5 ("Humanized Antibodies" or "Monoclonal antibodies").mp. [mp=title, book title, abstract,
 original title, name of substance word, subject heading word, floating sub-heading word,
 keyword heading word, organism supplementary concept word, protocol supplementary concept
 word, rare disease supplementary concept word, unique identifier, synonyms, population
 supplementary concept word, anatomy supplementary concept word] =115148

6 4 or 5 172688

7 Omalizumab.mp. or Omalizumab/ =4312

8 benralizumab.mp. =1059

9 r Interleukin-5/ or mepolizumab.mp. =1887

10 dupilumab.mp. = 4102

11 tezepelumab.mp. =321

12 Interleukin-13/ or lebrikizumab.mp. =6573

13 reslizumab.mp. or Interleukin-5/ =7029

14 (Xolair or MEDI-563 or MEDI 563 Z or Fasenra or BIW-8405 or SB-240563 or SB240563 or Nucala
 or Bosatria or SAR231893 or SAR-231893 or REGN668 or REGN-668 or Dupixent or tezspire or
 AMG-157 or MEDI-19929 or MEDI9929 or MEDI-9929 or ebglyss or RG-3637 or PRO301444 or
 MILR-1444A or MILR1444A or CEP38072 or CEP-38072 or DCP 835 or DCP-835 or SCH55700 or
 Cinqair or SCH-55700 or RHUMAB-E25 or lebrikizumab or TNX-650).mp. [mp=title, book title,
 abstract, original title, name of substance word, subject heading word, floating sub-heading word,
 keyword heading word, organism supplementary concept word, protocol supplementary concept
 word, rare disease supplementary concept word, unique identifier, synonyms, population
 supplementary concept word, anatomy supplementary concept word] =650

15 7 or 8 or 9 or 10 or 11 or 12 or 13 or 14 21439

16 6 or 15 =189440

17 Adolescent/ or Adolescent.mp. =2381574

18 (Adolescents or Adolescence or Youth or Teens or Teen or children or Teenager or Teenagers or
 Youths or Pediatric).mp. [mp=title, book title, abstract, original title, name of substance word,
 subject heading word, floating sub-heading word, keyword heading word, organism
 supplementary concept word, protocol supplementary concept word, rare disease
 supplementary concept word, unique identifier, synonyms, population supplementary concept
 word, anatomy supplementary concept word] =1824297

19 3 and 19 and 24 1339
